# Mechanical pretreatment of lignocellulosic biomass toward enzymatic/fermentative valorization

**DOI:** 10.1016/j.isci.2022.104610

**Published:** 2022-06-16

**Authors:** Carlos Arce, Lukas Kratky

**Affiliations:** 1 Czech Technical University in Prague, Faculty of Mechanical Engineering, Technická 4, Dejvice, 160 00 Prague 6, Czech Republic

**Keywords:** Biomass, Biotechnology, Chemical Engineering, Engineering

## Abstract

Lignocellulosic biomass (LCB) has the potential to replace fossil fuels, thanks to the concept of biorefinery. This material is formed mainly by cellulose, lignin, and hemicellulose. To maximize the valorization potential of this material, LCB needs to be pretreated. Milling is always performed before any other treatments. It does not produce chemical change and improves the efficiency of the upcoming processes.

Additionally, it makes LCB easier to handle and increases bulk density and transfer phenomena of the next pretreatment step. However, this treatment is energy consuming, so it needs to be optimized. Several mills can be used, and the equipment selection depends on the characteristics of the material, the final size required, and the operational regime: continuous or batch. Among them, ball, knife, and hammer mills are the most used at the laboratory scale, especially before enzymatic or fermentative treatments. The continuous operational regime (knife and hammer mill) allows us to work with high volumes of raw material and can continuously reduce particle size, unlike the batch operating regime (ball mill).

This review recollects the information about the application of these machines, the effect on particle size, and subsequent treatments. On the one hand, ball milling reduced particle size the most; on the other hand, hammer and knife milling consumed less energy. Furthermore, the latter reached a small final particle size (units of millimeters) suitable for valorization.

## Graphical abstract

**Figure:**
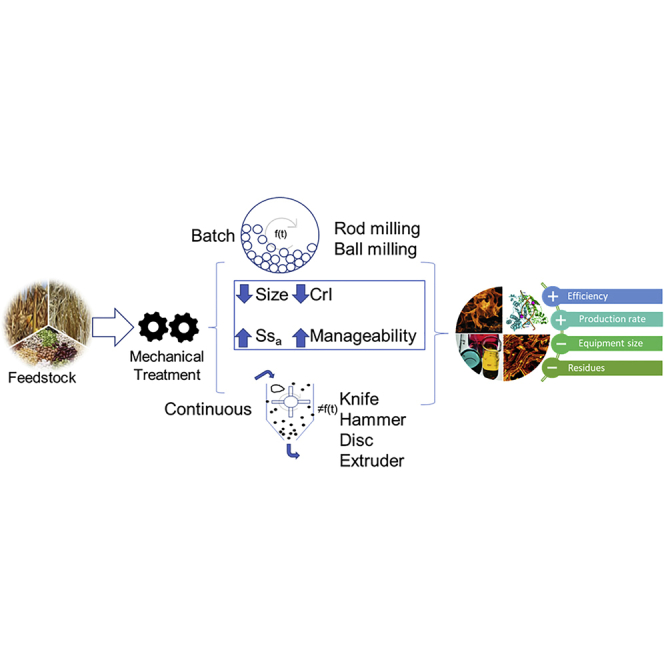

## Introduction

The growth of the global population, which is expected to reach 8.5 billion in 2030 (De Bhowmick et al., 2018), has led to an increase in energy use, and fossil fuels are one of the primary sources of energy. Thus, its usage has increased as well. Energy consumption is expected to rise a 50% from 2020 to 2050 (Office of Energy Analysis, 2019). Therefore, in recent years, environmental concerns and the scarcity of fossil fuels have resulted in the search for alternative energy sources (Danso et al., 2022; Silva et al., 2022). Biofuel is one of the alternatives for the replacement of fossil fuels. In fact, according to the International Energy Agency (IEA), the global demand for biofuels is expected to increase a 28% from 2021 to 2026 (IEA, 2021). LCB is the most used raw material for biofuel production. Therefore, it has become of interest as an environmentally friendlier way to obtain energy, chemicals, and bioplastics, when compared to fossil fuels, and has the potential to replace the exploitation of this kind of resource (Khoo et al., 2020), having a positive impact on the environment (Gundupalli et al., 2022). LCB comprises every plant and tree, either from forestry, agriculture, or as residue, and it is formed mainly by cellulose, hemicellulose, and lignin. Furthermore, it is renewable, biodegradable, and available (Guiao et al., 2022). Additionally, LCB can have zero net CO_2_ emissions (Bastidas et al., 2022; de Freitas et al., 2021) as trees and plants act as CO_2_ sinks (Saifuddin et al., 2020).

**Figure 1:**
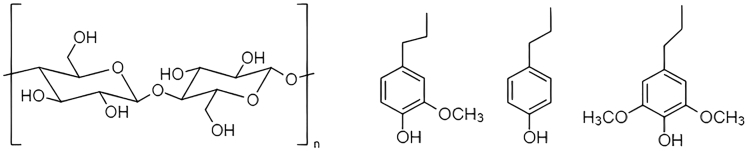
Cellulose monomer and lignin precursors (from left to right: Guaiacyl, hydrofenyl and syringyl)

Cellulose is a homopolymer formed by units of glucose linked together by a β 1,4-glycosidic bond, and it is the most abundant natural polymer on earth (Arce et al., 2020). Hemicellulose is a heteropolymer formed mainly by five-carbon sugars (Joy and Krishnan, 2022). Sugars can be obtained from both compounds and can be further used to produce biofuels, bioplastics, and other value-added chemicals. Lignin is another biopolymer, but it is formed by aromatic compounds, more specifically: *p*-hydrofenyl, guaiacyl, and syringyl units (Dou et al., 2021), and it is a source of antioxidants (Xiao et al., 2021). Figure 1 shows the precursors of lignin and cellulose monomer.

These compounds are located in the plant cell wall; however, they are interlinked, forming a crystalline-like structure that makes it recalcitrant and hinders sugar release (Soltaninejad et al., 2022). Therefore, LCB needs, in most cases, to undergo pretreatments to increase the accessibility of cellulose and, thus, increase the yield of the final processes (Sumiati et al., 2021).

These pretreatments can be classified as chemical, thermal, mechanical, and biological, depending on the driving force of the pretreatment (Zhao et al., 2022). Additionally, they can be combined. Although they are very different, the objective is: to break down the structure to expose cellulose and hemicellulose, eliminating lignin (Usmani et al., 2020). Chemical treatments eliminate lignin through solubilization and cleavage of the links that bound cellulose and lignin (Costa et al., 2018). However, they can generate some undesired products that could hinder the performance of the posterior biological/enzymatic treatments (Rasmussen et al., 2014). Thermal treatments are environmentally friendlier because there is no use of chemical products. Nevertheless, they can also lead to the formation of inhibitors, and it uses energy (Sidana and Yadav, 2022).

Biological treatments have low operating costs, and they can be easily installed. However, the efficiency is not high enough. Every treatment previously mentioned uses mechanical treatments as a preliminary technological step. This pretreatment uses mechanical force to break the primary cell wall, making cellulose more accessible to posterior treatments. As a result, specific surface area increases, and crystallinity decreases (measured by the crystallinity index: CrI), which overall increases the efficiency of the final process. The importance of the pretreatment is critical as the improvement of the following process would lead to a reduction in the equipment size, residues, and residence time. In addition, they can reduce lignin content (Kim et al., 2018) and increase its accessibility.

Additionally, they have a very high yield regarding inlet and outlet mass flow. This method works better with low lignin herbaceous plants; however, it can be used with woody materials and other lignocellulosic materials such as cellulose pulp (Tian et al., 2014; Zhou et al., 2018). This technology’s most significant disadvantage is energy consumption and low selectivity (Bychkov et al., 2019). As mechanical methods work incredibly well when herbaceous plants are used, which usually come from residues of other activities, it can be used as an alternative to the circular economy through a biorefinery concept, avoiding the most concerning issue regarding LCB: the use of cultivating land (Rulli et al., 2016), which can also be overcome by using microalgae (Chew et al., 2017).

Pretreated biomass can be valorized through several paths, depending on the final use: Chemically, enzymatically, fermentative, and thermally. The chemical path would aim to obtain chemical compounds mainly from the degradation of sugars contained in the cellulose matrix (Ji et al., 2018b); enzymatic and fermentative treatments can be performed separately (Ezeilo et al., 2017); however, they are usually performed together (Chen et al., 2015, 2016). Initially, enzymatic treatments aim to concentrate sugars so the fermentation process can increase its yield as glucose would be the substrate for the microorganisms to grow (Vasic et al., 2021). Finally, the objective of thermal treatments is generally to either obtain biochar (Rozenfelde et al., 2017) or products derived from pyrolysis (Liang et al., 2021).

This work recollects information on several types of equipment used to perform mechanical size reduction at a laboratory scale. It also shows the effect on particle size and the improvement of the following treatment. This information helps decide which kind of mill to use depending on the material, particle size required, and final valorization option. Additionally, it includes the authors’ critical review with considerations regarding scale-up from lab scale to industrial scale.

## Common mechanical treatments for size reduction of Lignocellulosic biomass

**Figure 2:**
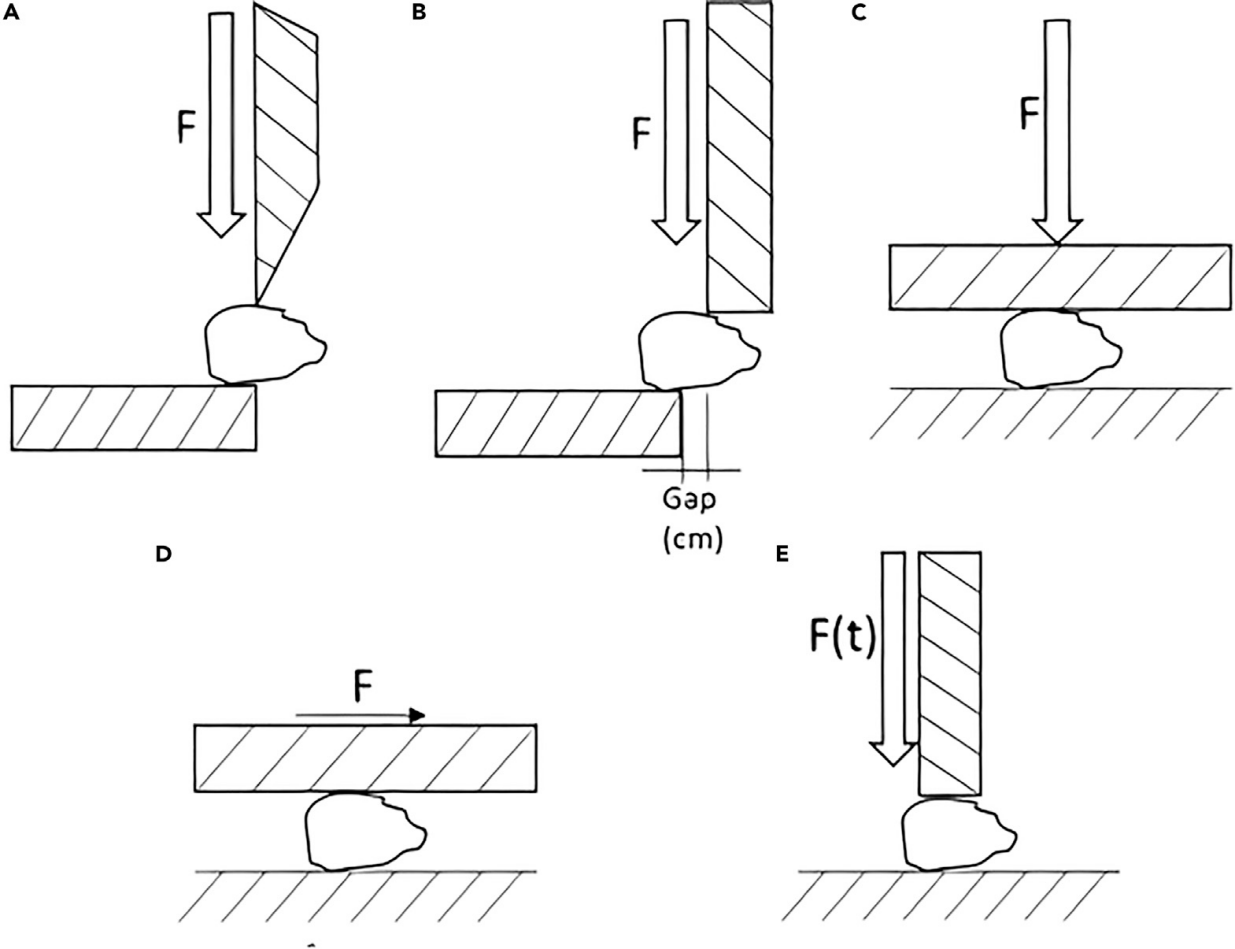
Illustration of the different size reduction mechanisms (A–E) (A) cutting, (B) shearing, (C) compression, (D) tearing, and (E) breaking.

In this section, the mechanical pretreatments performed on LCB are explained. Mechanical treatments are classified depending on the size reduction mechanism, generally applied to the biomass by an external body. When considering lignocellulosic biomass (LCB), cutting, shearing, compression, tearing, and breaking are the main mechanisms. Figure 2 shows a schematic description of the different mechanisms.

• Cutting: This mechanism occurs when the comminution machine has a sharp end.
• Shearing occurs between flat surfaces: one is fixed and the other moving. Usually, a gap is left between the two parts, so the comminuted material cannot pass through until adequate particle size is obtained.
• Compression crushes de material with continuous vertical force. This mechanism is more suitable to be used for brittle material.
• Tearing occurs when the moving part slides horizontally on the LCB and against the non-moving part.
• Breaking uses dynamic compression force to comminute biomass.

**Figure 3:**
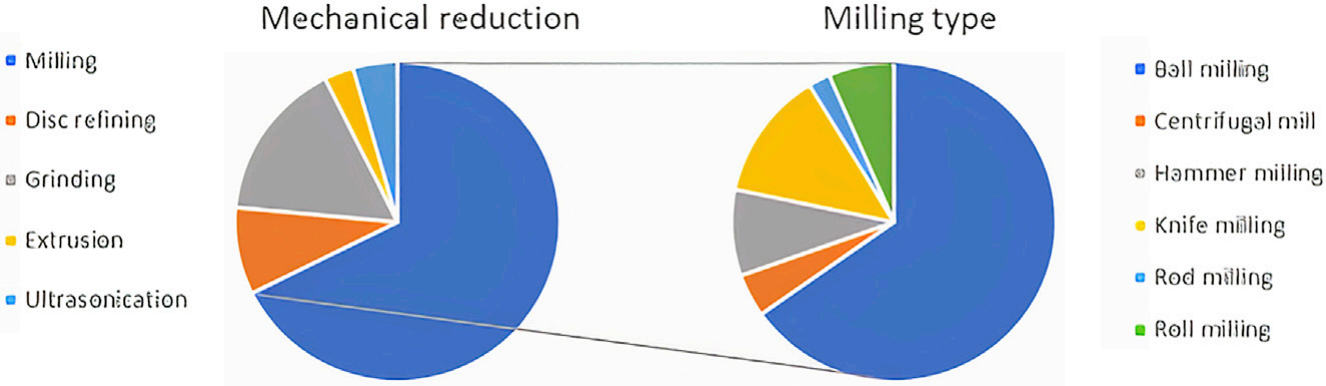
Mechanical treatments (left side), from most common to least common Milling (68%), grinding (16%), refining (9%), ultrasonication (4%) and extrusion (3%). Milling types (right side), from most common to least common: Ball milling (65%), knife milling (13%), hammer milling (9%), rod milling (7%), centrifugal milling (4%), and roll milling (2%).

Figure 3 shows the different mechanical pretreatments found in the bibliography in recent years, from 2019 to 2022, mainly from the Scopus database. This search was initially performed using the following keywords: Mechanical treatments, Particle size reduction, Mechanical pretreatment biorefinery, and Ball milling. These keywords gave the result of more than 130 articles. After eliminating those articles that did not fall into the topic, the number of articles was 67 (without considering review articles). Some references from further years are also included as they were interesting.

**Figure 4:**
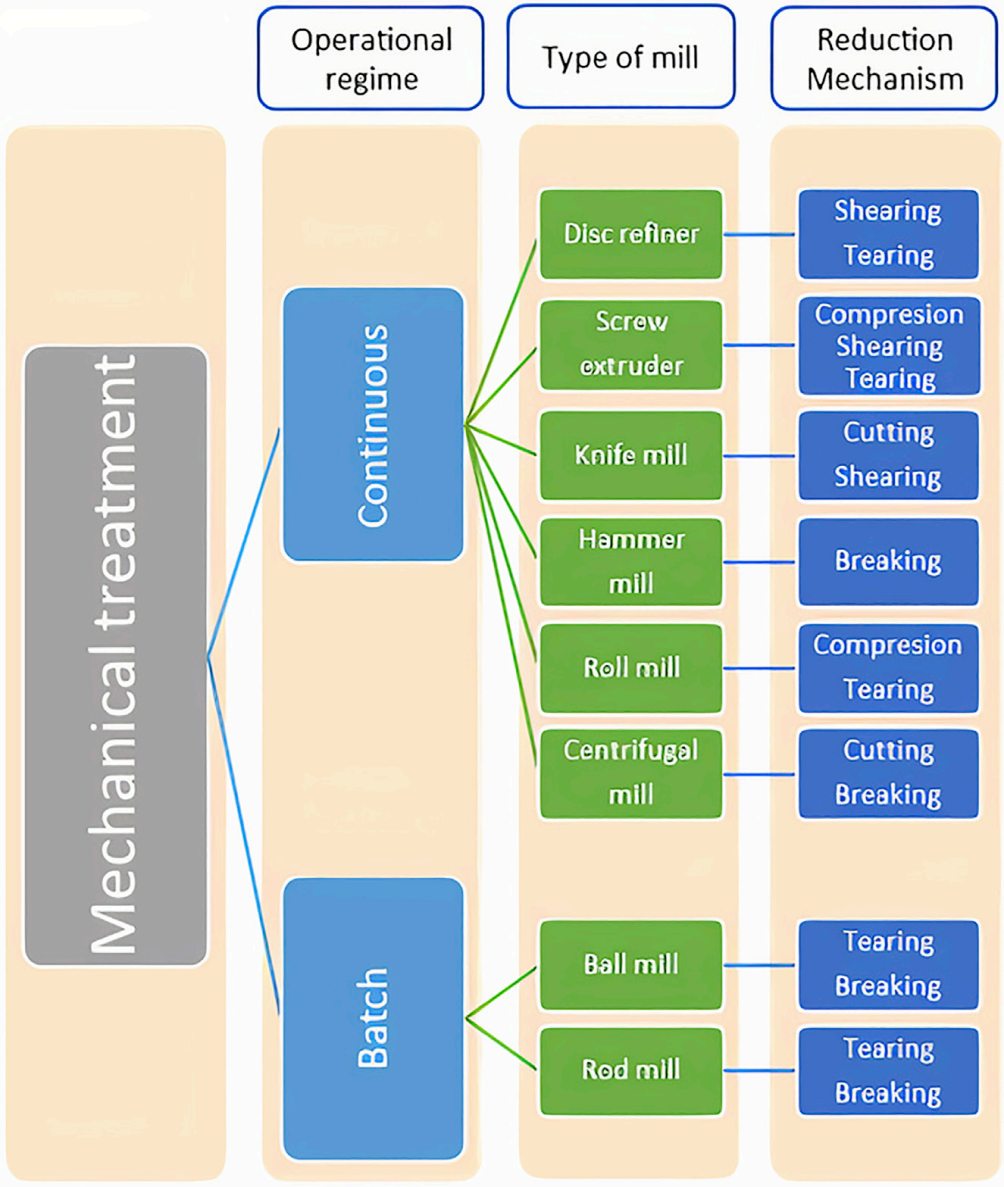
Classification of the different types of mills found

As can be seen from Figure 3, milling is the most used technique among the references found by the authors (68%), followed by grinding (16%), refining (9%), ultrasonication (4%), and extrusion (3%). Furthermore, among the different milling options, the most used type of mill is ball milling (65%), followed by knife milling (13%), hammer milling (9%), rod milling (7%), centrifugal mill (4%), and roll milling (2%). Figure 4 shows the different types of mills based on the primary reduction mechanism.

The classification from Figure 4 is further explained in the next part of the article. However, this illustration helps to understand the differences between the mills.

**Figure 5:**
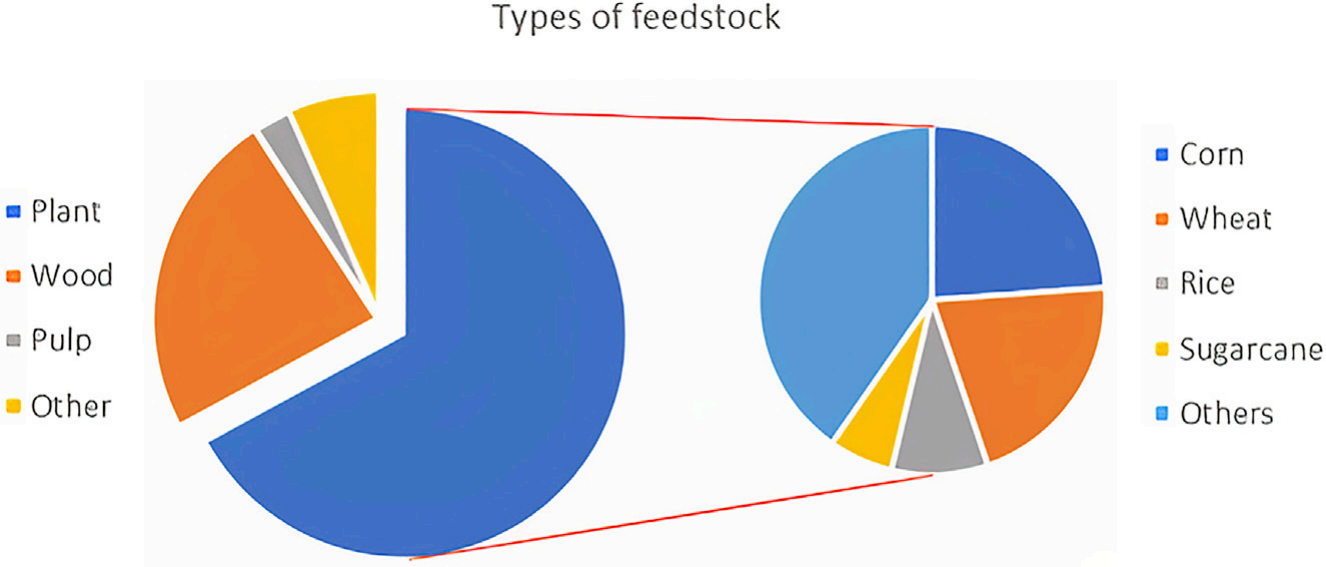
Feedstock used for mechanical reduction Plant (68%), wood (24%), Pulp (3%), and other (7%). Feedstock coming from plants (left side): Others include: corn, wheat, rice, and sugarcane.

Regarding the feedstock used by researchers in the bibliography consulted, LCB that comes from plants is the most used, specifically from agricultural and industrial wastes. Using this kind of feedstock shows the effort that researchers are making to valorize these residues and, as a result, become environmentally friendlier. Figure 5 shows the different biomass used as raw material.

Feedstock from plants is the most used for mechanical treatments. Residues from corn (24%) and wheat (21%) are widely used and account for 45% of all the residues used. Others from plants include grass, seeds, alfalfa cotton, and other residues. Sugarcane and rice residues accounted for 4% and 6%, respectively. As stated previously, LCB’s main compounds are lignin, hemicellulose, and cellulose. These compounds can be valorized into various products, from biofuels (through fermentation) to biochar (through thermal treatments). Additionally, the availability of this kind of material makes it an excellent option to be valorized.

## Equipment used for continuous milling

Mills that work under this configuration allow the material to go through the mill continually through the size reduction chamber in endless mode. It allows us to perform the size reduction with more quantity of raw material and, as a result, saves time and energy. Mills that work under this regime are explained next.

### Disc refiner

The equipment used for disc refining consists of two dented discs. One disc is static, thus not moving, and the other is connected to a rotor. Both discs are equipped with dented or specially shaped active size reduction tools. The main variables to control the final particle size are the disc’s rotational speed and the gap between the discs. Skinner et al. (2020) used this technology (aided with pressure) for the pretreatment and subsequent enzymatic hydrolysis of wheat straw. Biomass is fed into the center of the discs. Acceleration force moves biomass to the disc perimeter. Biomass radially flowing in disc gap is mechanically reduced in size between active working tools by shearing and tearing. The thinner the opening, the more intensive is the biomass size reduction.

**Figure 6:**
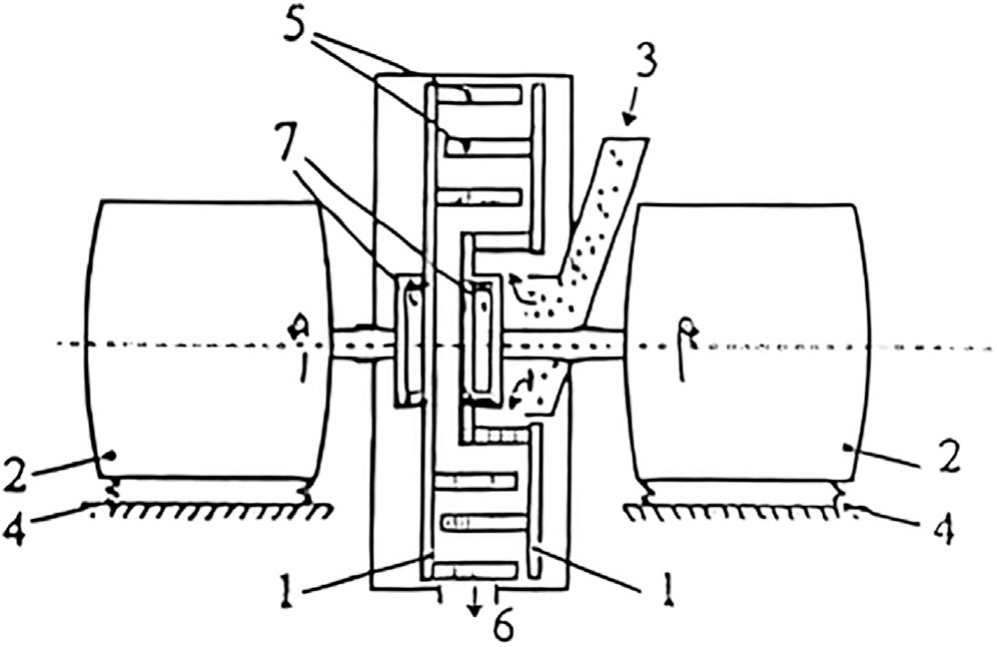
Disc mill (1) profiled rotors, (2) power drives, (3) material input, (4) elastic beds, (5) grinding elements, (6) output, and (7) adaptive controller.

Nevertheless, the high energy dissipation rate is usually recognized that can thermally degrade biomass components. Moreover, the disc gap also often tends to be plugged in case of fibrous and wet materials milling. It is, therefore, suitable to comminute dry biomass. Figure 6 shows an example of a disc refiner (Kratky, 2020).

The disc refining pretreatment leads to negligible acetic acid quantities and increased sugar release (Skinner et al., 2020). Ma et al. (2021) implemented disc refining as a pretreatment to produce biomethane from corn straw. The size reduction process increased the biomethane yield from 175.63 to 193.12 (L/kg). Furthermore, when the acidogenesis liquid phase was added to the biomass before size reduction, biomethane yield increased by 43% (Ma et al., 2021). This method can also be applied to produce biochemicals by pyrolysis treatment. It was demonstrated that after disc refining, pyrolysis led to a higher quantity of sugars and a metal ion reduction when compared to the raw material, leading to an improved pyrolysis product with no agglomeration of char (Torr et al., 2020). From the bibliography consulted, particle size after this operation is usually around 700 μm (Chen et al., 2019, 2020). However, if a smaller size is needed, other authors have reached particle sizes of around 120 μm (De Assis et al., 2018; Kim et al., 2016). Table 1 shows the references consulted on disc refiner as comminution process.

**Table 1:** References consulted on disc refiner

Disc refiner			
Final Treatment: Biological			
Feedstock	Conditions	Results	Ref
Prehydrolysis kraft pulp (PHK)	10% consistency, beating degrees from 19 to 50	Increased fock reactivity up to 78%, increased cellulase adsorption up to 71.2% CrI^a^ reduced from 68.8% to 47.1%	(Wang et al., 2020)
Corn stalk	Several methods	CrI^a^ reduced (34.4%, 29.3%, 28.7%) Increase of the release of xylan at a low concentration of NaOH (6%)	(Liu et al., 2021)
Alfalfa	Gap from 1 mm to 0.15 mm	Increase enzymatic hydrolysis by 8.2% (gap 1 mm)	(Chen et al., 2020)
Corn stover	Gap from 1 mm to 0.15 mm	Increase enzymatic hydrolysis by 36.4% (gap 1 mm)	(Chen et al., 2020)
White birch	Gap from 1 mm to 0.15 mm	Increase enzymatic hydrolysis by 5.4% (gap 1 mm)	(Chen et al., 2020)
Black spruce	Gap from 1 mm to 0.15 mm	Increase enzymatic hydrolysis by 3.6% (gap 1 mm)	(Chen et al., 2020)
Eucalyptus sawdust	0.5 mm gap, 5000 rpm	Increase enzymatic hydrolysis to 51%, glucan conversion increase to 52%	(Guigou et al., 2019)
White birch	Gap from 1 mm to 0.15 mm	Best conditions 8% consistency, 0.8 gap mm, improved sugar yield by 35%, and reduced specific energy consumption by 62%	(Chen et al., 2019)
Sugarcane bagasse	Disc gap = 0.002 and 0.005 in	Increased autohydrolysis of biomass from 69.6 to 77.2%	(De Assis et al., 2018)
Wheat straw	15, 30, 44 min, 4, 6, 8, 10 bar, two different discs	Enzymatic hemicellulose hydrolysis increased from 15% (non-pressure) to 35% (10 bar) Enzymatic cellulose hydrolysis increased from 30% (non-pressure) to 60% (10 bar)	(Skinner et al., 2020)
Corn straw	ND^a^	Under best conditions, biomethane yield reached 239 mL/gTS (47.13% higher than non-treated biomass)	(Ma et al., 2021)
Olive pomace	Several methods	Highest methane production: sieving<0.9.>Ball milling > Knife milling Highest energy requirements: Ball milling and ultra-fine grinding Sieving and Knife milling energy consumption could be compensated by biomethane production	(Elalami et al., 2018)
Napier grass	4, 10, and 40 mesh	Max. Methane production 4467.9 mLCH4/L, for 0.425 mm, 26 and 72% higher than for 2 and 4.75 mm	(Jomnonkhaow et al., 2021)
Napier silage	4, 10, and 40 mesh	Max. Methane production 3608.6 mLCH4/L, for 0.425 mm, 24 and 46% higher than for 2 and 4.75 mm	(Jomnonkhaow et al., 2021)
Wheat straw	1, 6, 8 and 10 bar, gap: 0, 15, 4 μm	During refining, cellulose reduction is negligible. Release cellulose twice	(Ward et al., 2021)

**Final treatment: Chemical**			

Corn stover	Minimal gap, 89 rpm	Disc milling increased sugar release for every scenario	(Kim et al., 2016)
Bioenergy sorghum	Min gap, 89 rpm	Maximum glucose and xylose release of 82.55% and 70.78%, respectively (pretreated at 190°C and 180º + disc milling)	(Cheng et al., 2019)

**Final treatment: Thermal**			

Plant waste	ND^a^	Increased HMF yield to 0.15% by microwave	(Zhou et al., 2021)
Pine wood	ND^a^	Reduction of metal ions and hemicellulose aided by the refining Increase in yield of pyrolysis sugars	(Torr et al., 2020)

a N.D., Non-Determined; CrI, Crystallinity Index; gTS, grams of Total Solids.

### Screw extruder

**Figure 7:**
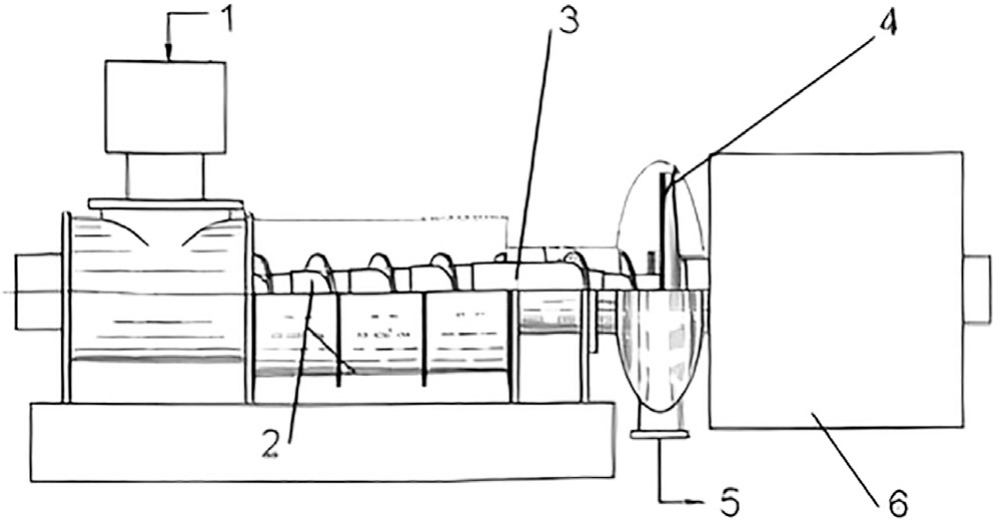
Extruder (1) input, (2) screw press, (3) decompression region, (4) colloid mill, (5) output, and (6) power drive.

Screw extrusion is an attractive technology for wet biomass processing, unlike disc refiner. Wet biomass is fed into a screw zone and transported to the opposite part of the screw. Biomass is mechanically reduced in size by shearing and tearing (Liu et al., 2013) in the gap between rotor and stator, or finally in the extrusion head. Friction forces rise in temperature; as a result, moisture content decreases and generates particle agglomeration, thus increasing mean particle size (Gu et al., 2019). Figure 7 shows an example of an extruder (Kratky, 2020).

Along the length of the screw, there can be openings so chemicals or any other treatment can be implemented and increase the efficiency of the pretreatment (Zheng and Rehmann, 2014). For example, Ämmälä et al. (2019) used a screw extruder for the sulfonation of pine sawdust to obtain microfibrilated cellulose. As a result, sawdust particle size was reduced and separated into fiber bundles, thus facilitating the obtention of microfibrilated cellulose (Ämmälä et al., 2019). Other authors used this technology to pretreat corn stalks to isolate xylan. It was found that using extrusion led to a higher xylan extraction than grinding but slightly lower than disc refining; the same trend was found for the CrI (Liu et al., 2021). Table 2 shows the references consulted regarding the screw extruder.

**Table 2:** References consulted on screw extruder

Screw extruder			
Final Treatment: Biological			
Feedstock	Conditions	Results	Ref
Corn stalk	Several milling methods	CrI^a^ reduced (34.4%, 29.3%, 28.7%) Increase of the release of xylan at a low concentration of NaOH (6%)	(Liu et al., 2021)
Wheat straw	17 rpm, counter-rotating screws	237 Nml methane/gVS^a^, Max daily production of 52 Nml methane/gVS^a^·day, 45% glucan yield	(Victorin et al., 2020)

**Final treatment: Other**			

Pine sawdust	85°C, 120 rpm	Production of microfibrilated cellulose from pine sawdust	(Ämmälä et al., 2019)

a CrI, Crystallinity Index; gVS, grams of Volatile Solids.

### Knife mill

A knife mill is a widely used size reduction machine to comminute dry biomass. Biomass is continuously fed into size reduction zones formed by static and rotor knife pairs. As material falls between the static and the rotating blade, it is cut and sheared. Comminuted biomass particles fall through a screen sieve to a balance storage tank. The driest and the most brittle the biomass, the highest is the dominance of the cutting principle. As biomass moisture increases, it becomes more elastic. Therefore, cutting effect dominance is reduced, and shearing becomes the dominant size reduction principle.

**Figure 8:**
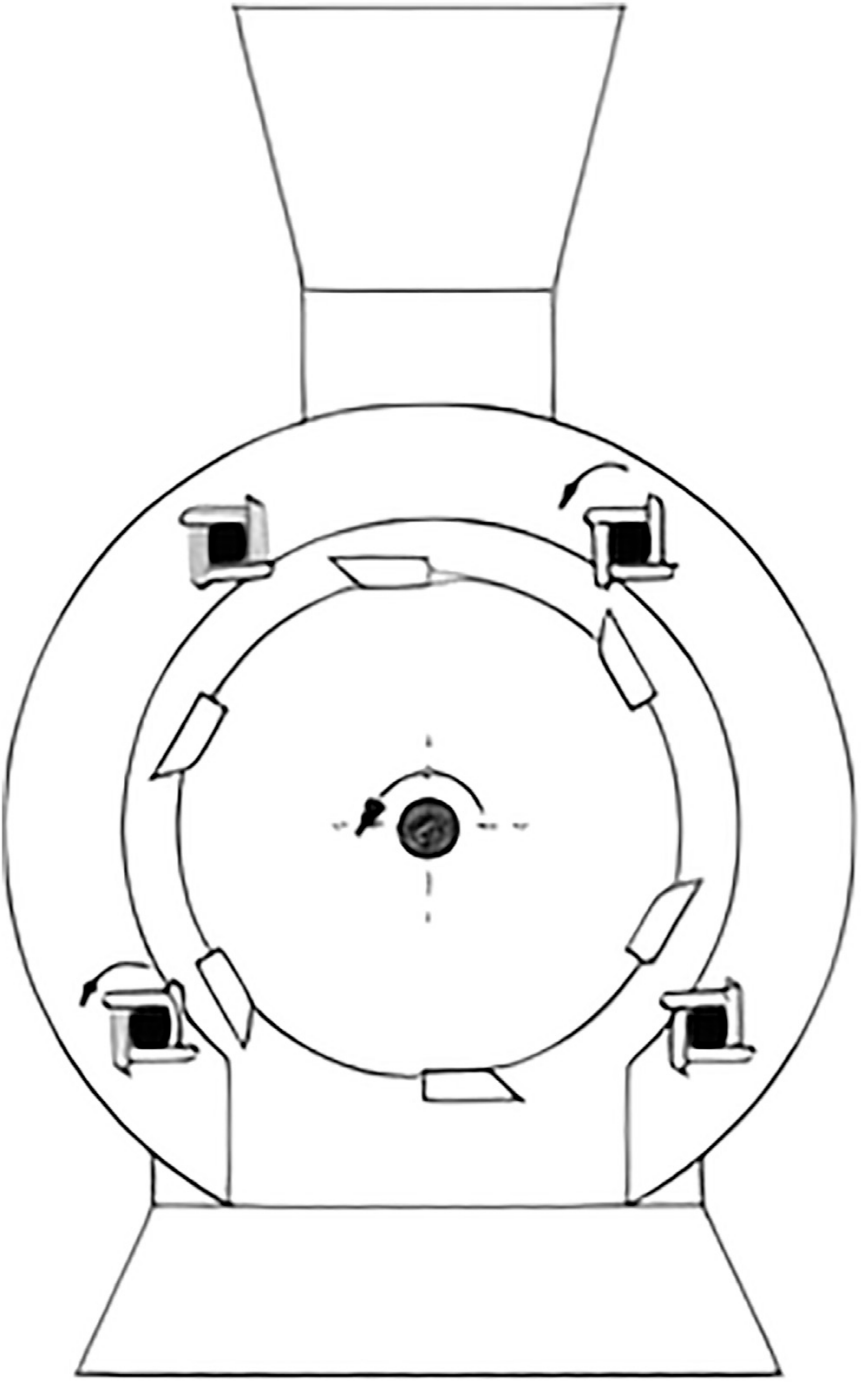
Milling chamber of a knife mill

Additionally, moisture makes biomass sticky. As a result, the size reduction efficiency is reduced owing to its effect associated with clogging screen sieve. Nevertheless, this kind of mill has been used by many authors as it can be operated at a high production rate and is easily performed. Figure 8 shows the milling chamber of a knife mill (Kratky, 2020).

Garuti et al. (2022) used knife milling to pretreat mixed seeds for methane production. They found out that 99% of the particles bigger than 5 mm were reduced after milling. Additionally, a 13% on methane yield was obtained (Garuti et al., 2022). Chuetor et al. (2021) used size reduction to treat rice straw with NaOH and increase cellulose concentration from 34.57% to 66.83% (mg/g Dried Mass) (Chuetor et al., 2021). Knife milling allowed the rice straw to be processable and increased specific surface area. Bianchini et al. (2021) used knife milling as a pretreatment so feedstock could be further valorized into CH_4_. The authors used additional size reduction equipment to obtain the most suitable size to produce biogas in this article. It was found that fines (<300 μm) had the highest CH_4_ yield and purity (Bianchini et al., 2021). The typical final particle size after knife milling depends on the sieve installed, but from the references found, the size can be reduced to 100 μm (Garuti et al., 2022). Table 3 shows the articles consulted regarding the knife mill.

**Table 3:** References consulted on knife mill

Knife mill			
Final Treatment: Biological			
Feedstock	Conditions	Results	Ref
Mixed Seeds	33.73 kJ/Kg Flow rate 1.6 to 10 t/h	13% increase in specific methane yield and SSA^a^ from 3.35 to 9.68	(Garuti et al., 2022)
Rice straw	6-2 mm sieve Moisture content 10.35 mg/g RS	Increased enzymatic digestion a 53.8%	(Chuetor et al., 2021)
Olive pomace	Several milling methods	Highest methane production: sieving<0.9.>Ball milling > Knife milling Highest energy requirements: Ball milling and Ultra-fine grinding Sieving and Knife milling energy consumption could be compensated by biomethane production	(Elalami et al., 2018)

**Final Treatment: Chemical**			

Hemp Hurd	ND^a^	CrI^a^ decreased from 59% to 44.15%	(Bokhari et al., 2021)

**Final Treatment: Thermal**			

Pine sawdust	ND^a^	The smallest particles achieved the highest gasification yield	(Liao et al., 2021)

a N.D., Non-Determined; CrI, Crystallinity Index; SSA, Specific Surface Area.

### Hammer mill

**Figure 9:**
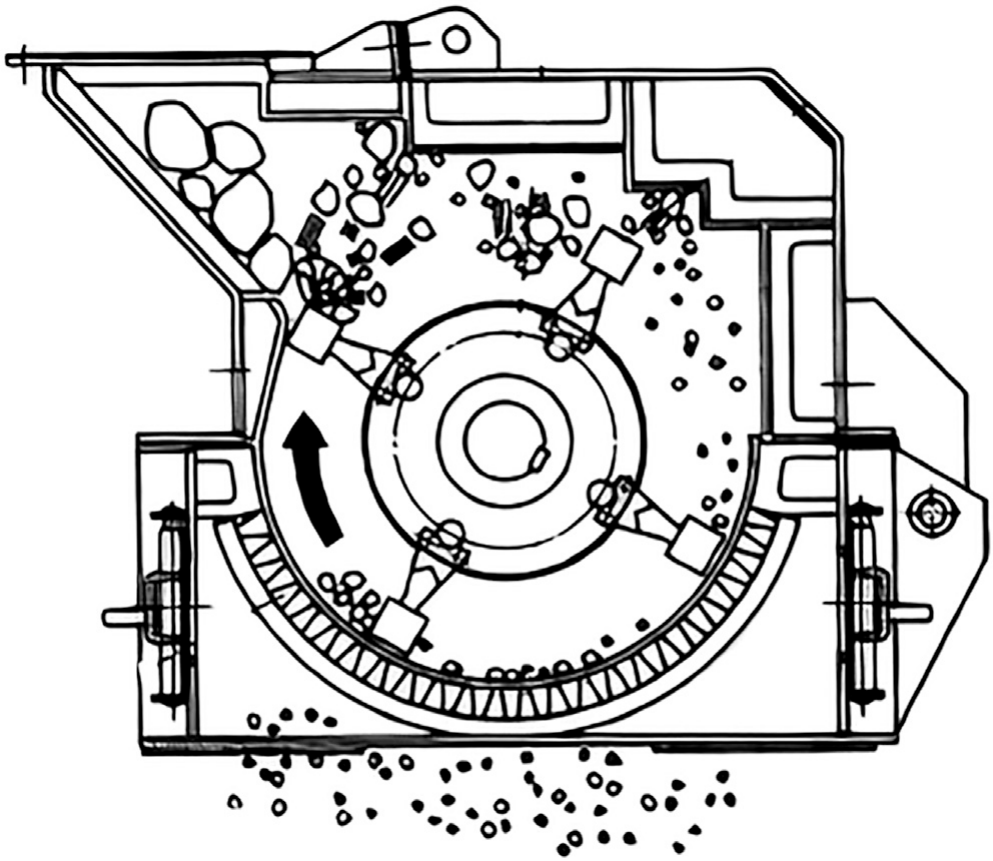
Hammer mill

Biomass enters tangentially to rotor hammers to receive a glancing impulse, dynamic effect of pressure force, to send it spinning toward a breaker plate, at which it is broke. Therefore, the primary mechanism responsible for the size reduction is breaking. As a result, comminuted biomass continuously and fractured pieces pass through a sieve. Regarding moisture, literature consulted showed that it has a negative effect on hammer milling, like knife mills. Furthermore, it is more harmful to hammer mills because of the size reduction mechanisms. Moisture makes biomass sticky, and it might adhere to the walls. As a result, these mills are more suitable for brittle material. Figure 9 shows the milling chamber of a hammer mill (Kratky, 2020).

Luo et al. (2021) used this technology to observe the influence of particle size on methane production. It was found that the sieve of 3 mm, where most of the particles ranged from 0.6 to 0.25 mm, was the most suitable size to perform the anaerobic digestion. It was not the highest methane yield (176.47 vs. 166.07 mLCH_4_/gVS for 1 and 3mm mesh, respectively). However, regarding energy consumption, it was the most efficient one. Size reduction also reduced the crystallinity of the biomass from 24.65 to 15.31% for the smallest particle size (Luo et al., 2021). Al Afif and Pfeifer, 2021 used hammer-milled cotton stalks to produce methane. It was found that the process increased the yield of biogas from 211 to 236 NL/Kg·VS and methane yield from 113.9 to 127.4 NL/kg·VS (Al Afif and Pfeifer, 2021). Particle size depends on the installed sieve, similar to knife mills. After comminution, the minimum particle found was 0.6 μm (Victorin et al., 2020). However, the most common final particle size ranged between 500 and 250 μm (Luo et al., 2021; Maitra and Singh, 2021). Table 4 shows the references consulted regarding hammer milling.

**Table 4:** References consulted on hammer mill

Hammer mill			
Final treatment: Biological			
Feedstock	Conditions	Results	Ref
Wheat straw	1 mm mesh	239 Nml methane/gVS^a^, Max daily production of 49 Nml methane/gVS^a^·day, 43% glucan yield	(Victorin et al., 2020)
Cotton stalks	ND^a^	Methane yield increased from 113.9 to 143.5 NL/KgVS	(Al Afif and Pfeifer, 2021)
Whole rice straw	1, 3, 5, and 7 mm	Reduced crystallinity from 24.65% to 15.31% (untreated material and smaller particles) Methane yield improved by 6.26%, 17.53%, and 27.65% (3, 5, and 7 mm sieve, respectively)	(Luo et al., 2021)

**Final treatment: Thermal**			

Energycane	Sieve 2 mm	Grinding increases sugar release than the untreated material Cryogrinding increased sugar release than traditional grinding	(Maitra and Singh, 2021)

a N.D., Non-Determined; VS, Volatile solids.

### Roll mill

**Figure 10:**
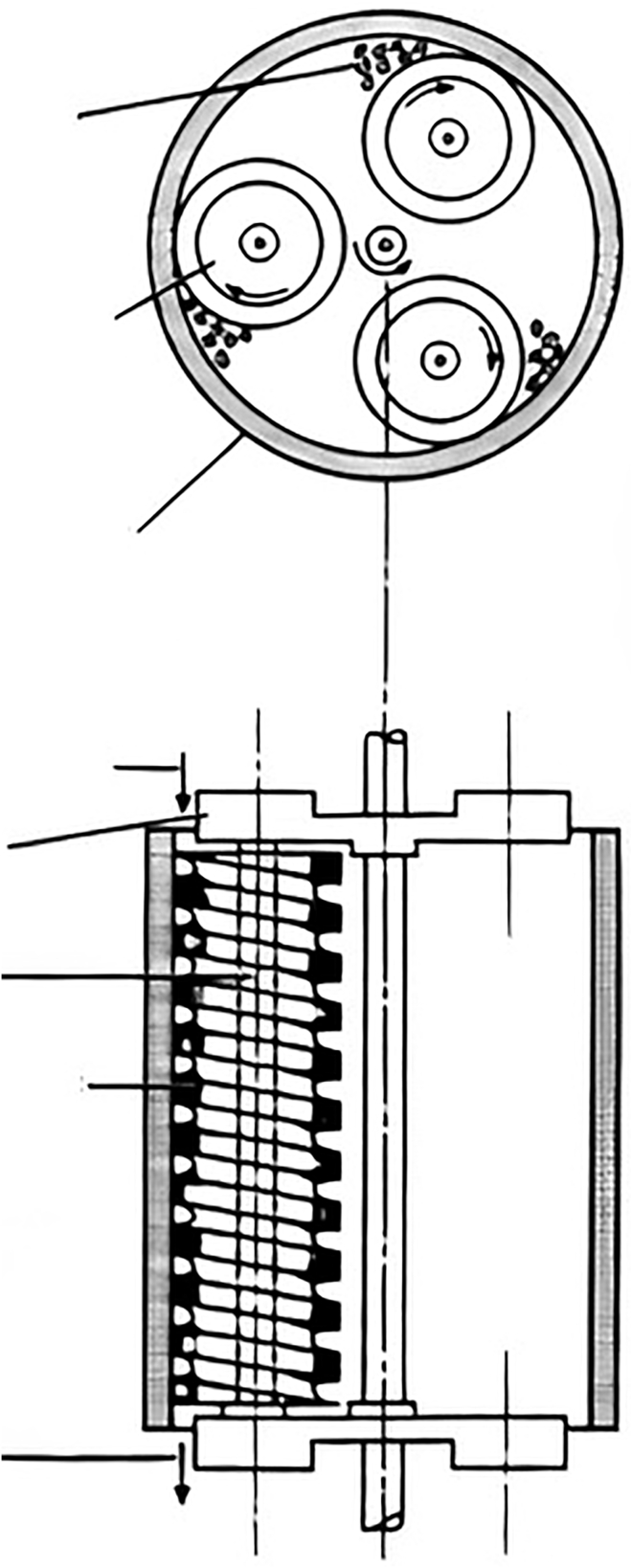
Scheme of a Szego mill (Chen et al., 2013) Parts from bot to bottom: Particle to be ground, helicoidal moving roll, shell, inlet for raw material, bearing assembly, roller shaft, Roller of hardened steel, outlet of grounded material.

Roll milling is used mainly for flour production, but it can also be used as pretreatment for the enzyme treatment of biomass. Roll milling consists of a series of opposed cylinders that can have a smooth or a dented surface. Biomass is comminuted continuously and steadily introduced between rolls. Thus, compression and tearing are the primary mechanisms of biomass particle size reduction. Roll mill is applicable only for the comminution of brittle or fibrous biomass. Wet biomass is sticky and usually adheres to the surface of rolls. These rolls are continuously spinning in different directions, and biomass is crushed between the gap of opposed cylinders (Cappelli et al., 2020). Finally, a unique roll milling equipment has been used by other researchers: Szego mill. It is similar to roll milling because it has a moving and a static part (Chen et al., 2016). The moving part is shaped like a screw, and the fixed part is the case. Figure 10 shows a scheme of a Szego mill (Chen et al., 2013).

Inside the case are more than one screw-like cylinders that rotate around its axis and the axis of the cage. Raud et al. (2020) used this technology to pretreat barley straw with three different approaches: dry, wet, and liquid nitrogen assisted. Dry milling obtained the highest BMP (from 269 to 292 L CH_4_/kg raw material). However, the highest production rate was obtained with liquid nitrogen assisted (Raud et al., 2020). The lowest particle size obtained from the bibliography was 17.25 μm (Bai et al., 2020).

Regarding roll milling, Bojanić et al. (2021) studied and optimized the roll milling process for wheat flour production regarding yield and energy consumption (Bojanić et al., 2021). However, this technology has been used for wheat production and to increase biorefinery options for biomass. For example, Victorin et al. (2020) used roll milling to reduce the particle size of wheat straw, and as a result, it increased the biogas potential of the biomass. As a result, the generated biochemical methane potential rose from 237 to 287 NmL CH_4_/g VS, the highest among the size reduction treatments studied by the authors (Victorin et al., 2020). Furthermore, other authors have used a particular kind of roll milling by changing the surface of one of the cylinders (Tsapekos et al., 2018) to use grass for biogas production, increasing CH_4_ yield from 305 mL/gVS to 367 mL/gVS and from 297 to 376 mL/gVS for both configurations. Table 5 shows references consulted on the roll mill.

**Table 5:** References consulted on roll mill

Roll mill			
Final treatment: Biological			
Feedstock	Conditions	Results	Ref
Corn stalk and sugar cane bagasse	Wet milling	Increased enzymatic digestion up to 33.09% (60-80 mesh) CrI^a^ from 45.81% to 57.53%	(Deng and Li, 2021)
Wheat straw	ND^a^	287 Nml methane/gVS^a^, Max daily production of 41 Nml methane/gVS^a^·day, 34% glucan yield	(Victorin et al., 2020)
Different kinds of grass	ND^a^	Methane yield from 474 to 299 mL/gVS^a^ (untreated 33.9 mL/gV^a^S)	(Tsapekos et al., 2018)
Grass clippings	ND^a^	Methane yield 326 mL/gVS^a^ (untreated 33.9 mL/gVS^a^)	(Tsapekos et al., 2018)
Wheat straw	ND^a^	Methane yield 255 mL/gVS^a^ (untreated 33.9 mL/gVS^a^)	(Tsapekos et al., 2018)
Digested biofibres	ND^a^	Methane yield 42 mL/gVS^a^ (untreated 33.9 mL/gVS^a^)	(Tsapekos et al., 2018)

a N.D., Non-Determined; CrI, Crystallinity Index; gVS, grams of Volatile Solids.

### Centrifugal mill

**Figure 11:**
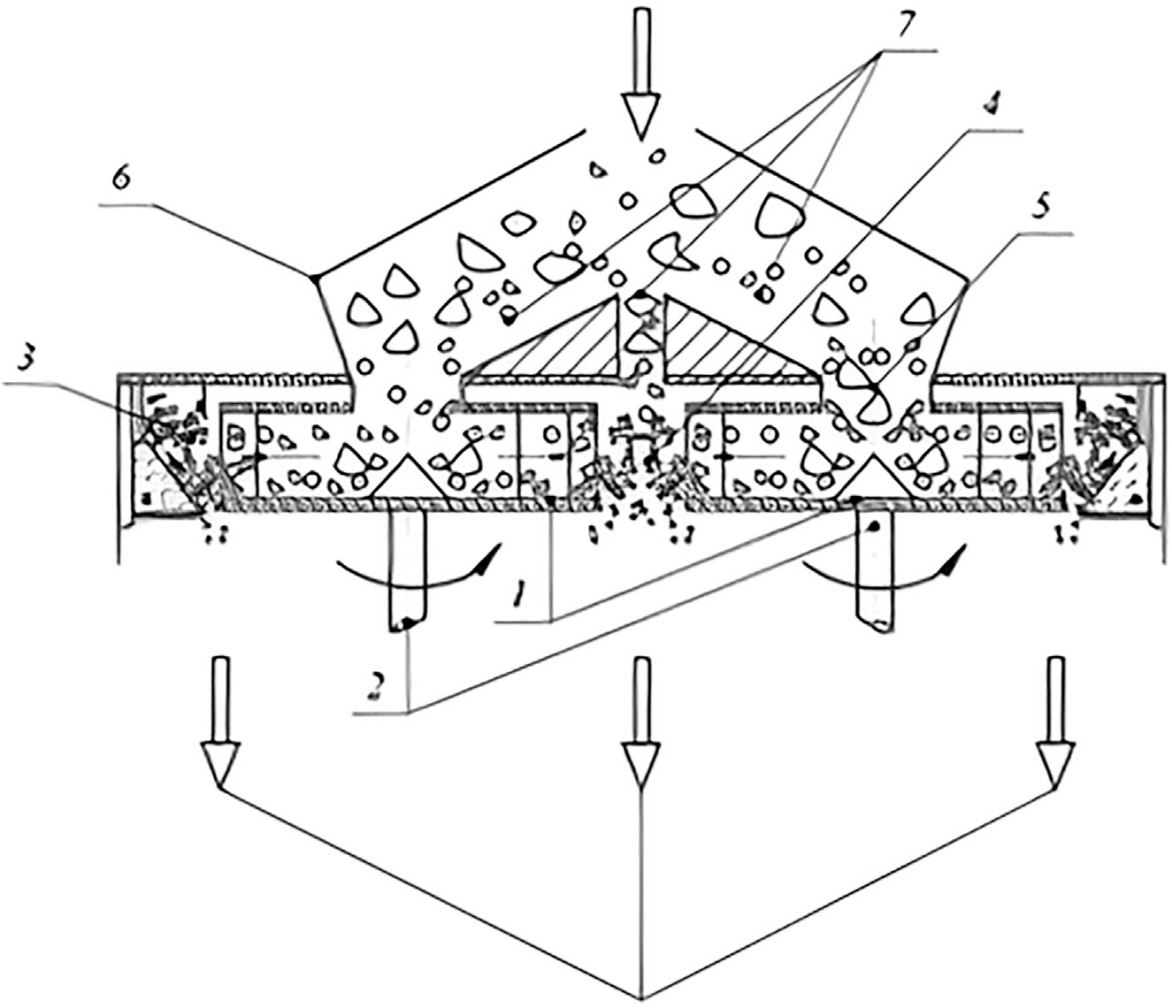
Example of a centrifugal mill (Nadutyi et al., 2019) (1) rotor, (2) rotating shaft, (3) disintegration chamber, (4) central gap, (5) feed channel, (6) multichannel boot device, and (7) main channels. Under creative commons license.

This type of mill uses high rotational speed to reduce the size of the material. The material enters the mill from the upper part. Inside the milling chamber, there is a rotor with blades that spins at high speed, thus the size reduction mechanisms are cutting and breaking. When the biomass enters the chamber, the mill’s blades cut the material, and the resulting particles are thrown at the mill’s walls. The mill wall has a mesh that keeps the particles inside until their size is small (Fernando and Manthey, 2022). Figure 11 shows a schematic representation of a centrifugal mill.

Ivanchenko et al. (2021) used this comminution technology to treat a mixture of vegetable waste with sewage sludge to produce biogas. It was found that fermentation time decreased from 25 to 12 days after comminution, and biogas production increased by 41% (Ivanchenkoet al., 2021). Other authors used this process to increase the solid load of biomass for bioethanol production. Using the smallest size (≤2.5 mm) had a 16.9% yield glucose concentration, higher than when using the largest size. Additionally, it was discovered that increasing solid loading from 10 to 35% led to a 460% increase in glucose concentration (Hoppert and Einfalt, 2021). Table 6 shows the references to the centrifugal mill.

**Table 6:** References consulted on centrifugal mill

Centrifugal mill			
Final Treatment: Biological			
Feedstock	Conditions	Results	Ref
Rice straw	Ambient temperature, 8% moisture content, 0.25 mm sieve	0.3921 g glucose/g biomass with 94% glucose conversion	(Areepak et al., 2022)
Corn stover	0, 2, 4, and 6 h	6 h grinding, with 150 mg/L nanomaterials Max yield 425 mL H2	(Tahir et al., 2021)
Vegetable residues and activated sludge	ND^a^	Particle size decreases from 50 to 16 μm-- > increases biogas yield by 30%. Particle size reduction and whey increased biogas yield by 41%	(Ivanchenko et al., 2021)
Wheat straw	ND^a^	Reduced particle size allowed to increase solid loads up to 35%. Increase glucose concentration a 460% compared to 10% solid load.	(Hoppert and Einfalt, 2021)

a N.D.: Non-Determined.

## Equipment used for batch milling

**Figure 12:**
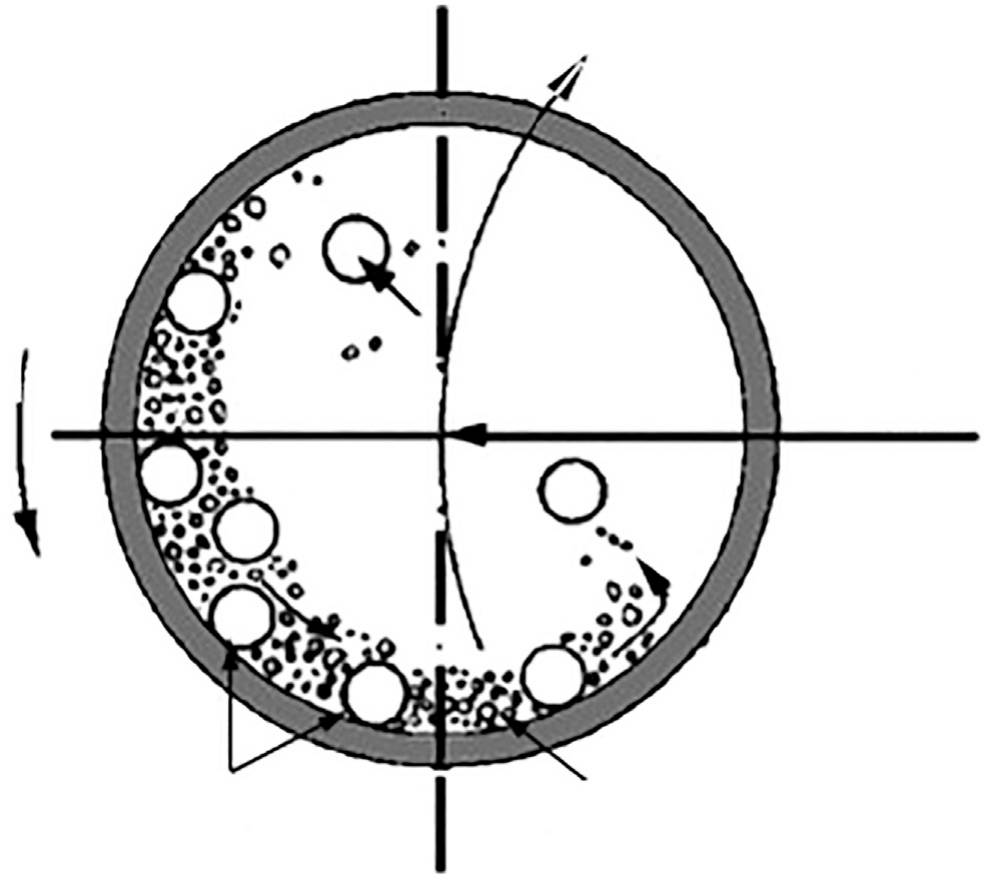
Scheme of a foreign body motion pattern in a single pot of a planetary mill Inside the milling chamber the set elements and the LCB. Big arrows show forces and rotation: Rotation of the milling vials (left), rotation of the supporting discs (in the middle), and centrifugal force (right).

The mills with freely set elements are batch size reduction machines that can be applied to comminute biomass independently of its moisture. The ball chamber is a crucial part of the mill. It is fed by the proper amount of biomass and grinding elements (steel spheres or rolls). The closed chamber with biomass and grinding parts starts to rotate. A cylinder slowly rotates and moves grinding elements, crushing biomass in the fall. Biomass is also comminuted by its friction between grinding elements and between elements and shells. These mills operate in batch mode, meaning that the final product does not continuously come out of the equipment. Figure 12 shows the milling chamber with the free elements and all the forces that intervene in the process (El-Eskandarany, 2015).

### Ball mill

The balls and the feedstock are introduced in a cylinder in a large ratio. Then, the cylinder starts moving, rotating around its central axis. The size reduction occurs by the friction forces between the cylinder and the balls and between the balls themselves. The mechanism responsible for the size reduction depends on the speed of the mill. If the speed is low, the grinding material roll over itself. Thus, the primary size reduction mechanism is tearing between the balls, feedstock, and mill wall. As speed increases, it reaches a point where the centrifugal force makes the grinding material reach the highest point inside the cylinder and fall under the influence of gravity. Thus, breaking becomes an additional mechanism responsible for size reduction. Finally, if the speed is high enough, the grinding material does not fall and distributes along the surface of the chamber. Thus, tearing becomes the only size reduction mechanism but is less efficient than low-speed abrasion. This effect is shown in picture 12. Generally, it is assumed that the optimal speed for size reduction is the one that makes the grinding material reach the maximum height inside the grinding chamber, so abrasion and impact are ensured (Lomovskiy et al., 2020).

Regarding the composition of the grinding material, most of the authors use zirconia (Ji et al., 2018a; Wang et al., 2021; Yu et al., 2019); however, steel (Wu et al., 2021), agate (Yang et al., 2022), aluminum oxide (Kobayashi et al., 2021) and others (Rajaonarivony et al., 2021), can be used. Over the last years, ball milling has been extensively used as size reduction pretreatment for biomass processing, not only because it is easy to perform but also because it can be used with chemicals (mechanochemical) to increase the efficiency of posterior treatments. For example, Xiao et al. (2020) used ball milling on bamboo residues to increase enzymatic saccharification, reducing the CrI of biomass from 71.3% to 9.5% (Xiao et al., 2020). On the other hand, Qi et al. (2019) used a mechanochemical treatment consisting of ball milling of rice straw and rice straw-derived black liquor catalyst. As a result, glucose and xylose yields of 52.1% and 66.5% were obtained, respectively (Qi et al., 2019). Lempiäinen et al. (2020) employed sulfuric acid and ball milling to observe the structural changes in willow. Additionally, a total reducing sugars yield of 14.8% was obtained compared to the 1.9% yield without mechanochemical treatment (Lempiäinen et al., 2020). Regarding particle size after milling, it was found that this technology can achieve tiny particle size. The lowest was 9.66 μm (Liu et al., 2019). However, the most common particle size obtained from ball milling was around 20 μm (Rajaonarivony et al., 2021), even when the initial particle size was very large (Navarro-Mtz et al., 2019).

### Rod mill

In this type of mill, the free set elements are rods. As the milling chamber rotates, the rods move, clashing with each other and the material. The main mechanisms responsible for the size reduction are abrasion and impact. Not many references regarding rod milling and biomass processing for enzymatic production have been found. However, Bai et al. (2020) used this technology as pretreatment for pyrolysis and biochar production. It was found that torrefaction + rod milling leads to the best results in bio-oil characterization and composition and morphological properties of the biochar (Bai et al., 2020). Table 7 shows the references consulted on ball and rod milling.

**Table 7:** References consulted on ball and rod mill

Ball mill			
Final treatment: Biological			
Feedstock	Conditions	Results	Ref
Corn stover	30°C, 30 min, Volume ratio 2:1	Increased enzymatic hydrolysis from 20.1 to 41.41 mg/g glucose yield Crystallinity reduced to 18.26%	(Yu et al., 2019)
Sugarcane bagasse	50°C, 60 min, 500 rpm	Saccharification increased from 10.3% to 34.8% and up to 79.7% (AlCl3) CrI^a^ from 52.8% to 22.0%	(Zhang et al., 2019)
Corn stover	5, 10, 20, 30, 60, 90 and 120 min, 30°C	Max CrI^a^ reduction 10.4%, Max yield of ethyl levulinate 53.55% (120 min and 180°C)	(Liu et al., 2018)
Wheat straw	5.53% moisture content, 30 and 60 min, 20°C	Min CrI^a^ 19.49%, 60 min ball milling increased glucose yield up to a 164.5%	(Ji et al., 2018a)
Rice straw	5.84% moisture content, 30 and 60 min, 20°C	Min CrI^a^ 20.05%, 60 min ball milling increased glucose yield up to a 120.9%	(Ji et al., 2018a)
Birch	450 rpm, 6 h	Min enzyme load (0.25 mL enzyme/g Biomass), <10% impurities. Two Cycles	(Wang et al., 2021)
Pine	450 rpm, 6 h	Min enzyme load (0.5 mL enzyme/g Biomass), <10% impurities. Two Cycles	(Wang et al., 2021)
Reed	450 rpm, 6 h	Min enzyme load (0.5 mL enzyme/g Biomass), <10% impurities. Three Cycles	(Wang et al., 2021)
Walnut shell	450 rpm, 6h	Min enzyme load (0.5 mL enzyme/g Biomass), <10% impurities. Three Cycles	(Wang et al., 2021)
Corn stover	B500 rpm, 1-3 h	Ball milling reduced the recalcitrant nature of LCB Optimal conditions lead to 69.65% xylo-oligosaccharides	(Zhang et al., 2021)
Wheat straw	1, 2, 3, and 4 h, 450 rpm	CrI^a^ reduced from 46% to 7.6%, Glucose yield increased up to 99.4% (delignification 79.2%)	(Liu et al., 2022)
Aspen	Enzymatic digestion assisted	Reduced enzymatic hydrolysis time from 72 h to 24 h and buffer solution, 84.7% glucose yield (24 h)	(Wu et al., 2021)
Cellulose + chitin	Enzymatic digestion assisted	Enzymatic digestion is boosted by mechanical forces rather than local heat	(Kobayashi et al., 2021)
Enset fibers	15, 30, 60, 90, and 120 min, 200, 350, and 500 rpm	Dry chemo-chemical treatments increased glucose yield to a max of 621.3 g Glucose/Kg raw material in 90 min	(Sitotaw et al., 2022)
Sugarcane bagasse + Pennisetum	2 h, 400 rpm (Assisted with NaOH solutions)	Bagasse max reducing sugar yield 40.75%, 4% NaOH, hydrothermal 100°C 40 min Pennisetum max reducing sugars 55.74%, 4% NaOH, hydrothermal 80°C 60 min	(Huang et al., 2019)
Soy bean meal	400 rpm and 2, 5, 8, 10, and 20 min	Best result at 5 min milling time, 34.1 times more sugars than untreated soybean and 2.5 times more sugar than commercially used soybean meal	(Navarro-Mtz et al., 2019)
Corn stover	10, 20, 30, 60, and 120 min, 20°C	Crystallinity reduced from 46.52 to 5.04 (120 min) Ball milling allowed enzymatic digestion at high solids load, max monomeric sugar concentration (120 min, 30% solids load, and 10 FPU)	(Lu et al., 2020)
Olive pomace	Several milling methods	Highest methane production: sieving<0.9.>Ball milling > Knife milling Highest energy requirements: Ball milling and ultra-fine grinding Sieving and Knife milling energy consumption could be compensated by biomethane production	(Elalami et al., 2018)

**Final treatment: Chemical**			

Wheat straw	600 rpm, 30–40°C, 2 h	Narrower size distribution reduced CrI^a^, higher hemicellulose and lignin removal, at <10% and <4% NaOH concentration, respectively	(Gao et al., 2021)
Wheat straw	NaOH-assisted, 600 rpm, 2 h, (0.5, 1, 2, 4, 6, 8, 10, 12 wt % NaOH)	Narrower size distribution, reduced CrI^a^, higher hemicellulose and lignin removal at high NaOH concentration	(Gao et al., 2021)
Willow sawdust	800 rpm	Milling time increased monosaccharides release CrI^a^ decreased from 59% to 14%	(Lempiäinen et al., 2020)
Corn stover	5, 10, 20, 30, 60, 90 and 120 min, 30°C	Milling time reduced CrI from 42.62% to 10.40% Maximum ethyl levulinate yield 53.55%@180°C, for 120 min milling	(Liu et al., 2018)
Peanut Shell biochar	300 rpm, 4,8 and 12 h	Increased H_2_O_2_ selectivity up to 87% Increased H_2_O_2_ rate 1.9 and 2.8 times when compared to pine cone shell and sawdust biochar	(Gao et al., 2022)
Bamboo residues	ND^a^	Longer times lead to increase isolation yields from 39.2% to 53.9%	(Yang et al., 2021)
Poplar sawdust	ND^a^	Longer times lead to increase isolation yields from 15.5% to 35.6%	(Yang et al., 2021)
Larch sawdust	ND^a^	Longer times lead to a minor increase in isolation yields from 23.4% to 25.8%	(Yang et al., 2021)
Rice husk	300 rpm, 20 or 30 min	Increased silica yield up to 89% and 6% (w/v) solid content and silica purity was increased to 98.5% No structural changes to the final product	(Park et al., 2021)
Corn stover	3, 5, 10, 20, 30, 60, 120, 240 and 480 min, 30°C	CrI^a^ was reduced from 76.91% to 7.62% increasing milling time Methyl levulinate yield increased to 58.12 mol % at 160°C in 60 min and 64.92 mol % at 170°C in 45 min	(Chen et al., 2019)
Rice straw	500 rpm, 4 h,	Ball milling + catalysts increased glucose and xylose yield (52.1% and 66.5%, respectively)	(Qi et al., 2019)
Mulberry wood	0-8 h	Ball milling increased the yield of succinoylation from 25.7% to 31.8% Max. Yield 65.8% when chemically assisted	(Chen et al., 2019)
Corn stover	5, 10, 20, 30, 60, 90 and 120 min, 30°C	Carbohydrate content in water extracts increased with milling time	(Liu et al., 2019)
Cellulose	350 rpm, 4 h	Crystallinity reduction from 77.1 to 48.1 (B.M.) and 43.4% (BM-Al) Increase 5-HMF yield to a maximum of 40% (B.M.) and 45% (BM-Al)	(Shen et al., 2020)

**Final Treatment Thermal**			

Hickory wood	300 rpm, 12 h	Positive effect on biochar rich in functional groups and good dye removal	(Yang et al., 2022)
Plant waste	150, 180, 210, 240 and 270 h, 300, 350, 400, 450, 500 rpm	Increased HMF yield to 1.8% (Max reached) by microwave	(Zhou et al., 2021)
Wheat straw	ND	Milling improved the adsorption capacity of wheat straw of biochar. Ball milling contributed to the precipitation of Pb(II)	(Cao et al., 2019)
Straw	60 rpm	The highest specific energy used, milling, was quicker on bark (brittle) than on straw (elastoplastic)	(Rajaonarivony et al., 2021)
Straw	330 rpm	Milling was quicker on bark (brittle) than on straw (elastoplastic)	(Rajaonarivony et al., 2021)
Bark	60 rpm	The highest specific energy used, milling, was quicker on bark (brittle) than on straw (elastoplastic)	(Rajaonarivony et al., 2021)
Bark	330 rpm	Lowest specific energy used, milling was quicker on bark (brittle) than on straw (elastoplastic)	(Rajaonarivony et al., 2021)
Flax	60, 140, 330, 420, 480, 600, 1020, 1380 min,	Reduction of crystallinity of. Increase accessibility of water, produced by the increase of the amorphous cellulose	(Mayer-Laigle et al., 2020)
Eucalyptus sawdust	Ultrasound	Obtention of nanocellulose	(Ferreira et al., 2020)

**Rod mill**			

Wheat straw	ND	CrI reduced to 11.59%, 24.12% when torrefied Increased bio-oil yield to 46.16%	(Bai et al., 2020)

a N.D., Non-Determined; CrI, Crystallinity Index.

## A critical overview of size reduction machines

The review summarized information about size reduction machines’ applications to reduce biomass particles and the effect of mechanical size reduction on process efficiency of subsequent technological pathways. Ball mill and disc refiners are the most conventional mechanical size reduction machines applied on a laboratory scale, followed by knife and hammer mills. Regarding ball or disc milling, it can be stated that their use allows us to reach biomass particle size in tents and even lower, hundreds of micrometers. Nevertheless, their application potential in industrial biorefineries is minimal.

• Ball mill is an advantageous batch size reduction machine allowing biomass comminution at any moisture. However, it shows the highest specific energy demand, around thousands kWh/t of biomass. In addition, its productivity is limited by the residence time of a given batch, typically in tenths of minutes (Kratky and Jirout, 2011).
• Disc refiner is the least reliable as biomass usually clogs the gap. When blocked, temperature increases because of heat dissipation, thus, potentially damaging biomass, especially wet and fibrous biomass. Therefore, it is best suitable for dry biomass. Specific energy demand usually meets the values of hundreds of kWh/t for straw-based biomass (Kratky and Jirout, 2011).

The balance between suitable particle size and subsequent process efficiency was studied in several reports. Biomass particle size between 0.03 and 10 mm is essential for fermentation (Oyedeji et al., 2020). Miao et al. (2011) present the need for 0.5-3.0 mm in corn stover for bioethanol production technology. Sharma et al. (1988) reported biomethane yields for 362 Nm3 t-1 T.S. for particles of 0.088 mm, 360 Nm3 t-1 T.S. for particles 0.40 mm, 350 Nm3 t-1 T.S. for particles 1 mm, 330 Nm3 t-1 T.S. for particles 6 mm a 235 Nm3 t-1 T.S. for particles of 30 mm. Regarding these results, particle size under 1 mm can be disadvantageous in fermentation process control. Izumi et al. (2010) found that when particle size is smaller than 1 mm, the hydrolytic microorganisms are intensively affected by the smallest particles. Lower fatty acids are formed rapidly during their degradation, the pH of the substrate drops sharply, and the methanogenesis process is inhibited. Regarding laboratory results, the biomass particles of units in mm seem to be a suitable particle size for biomass treated in industrial lignocellulosic biorefineries. Knife or hammer mills are, therefore, suitable mechanical size reduction machines. These machines ensure continual processing of biomass with moisture up to 25% w/w in high volumes under the least specific energy demands (Kratky and Jirout, 2011) being in units of tenths of kWh/t for straw- or wood-based biomass.

## Biomass final treatments

**Figure 13:**
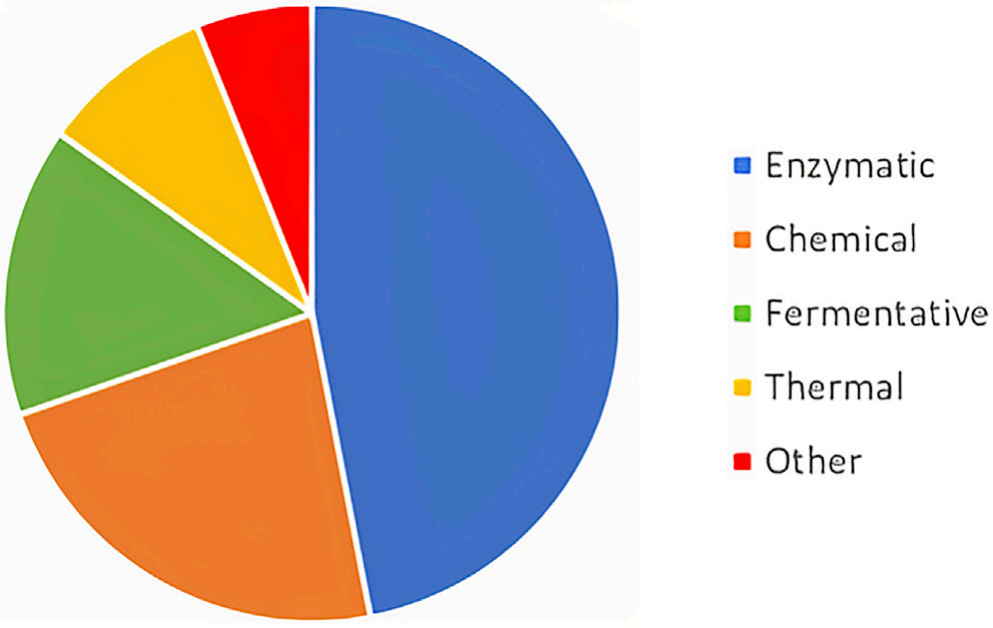
End-use treatments for biomass after mechanical treatments, found in bibliography from most common to least common Enzymatic (47%), chemical (23%), fermentative (15%), thermal (9%), and other treatments (6%).

The treatments mentioned above are usually coupled with the final treatment. From the literature revised by the authors, most of these end-user processes focus on biofuels and biogas. Thus, fermentative and enzymatic processes are the most common end-use processes. Figure 13 shows the end-use processes after mechanical pretreatment.

**Figure 14:**
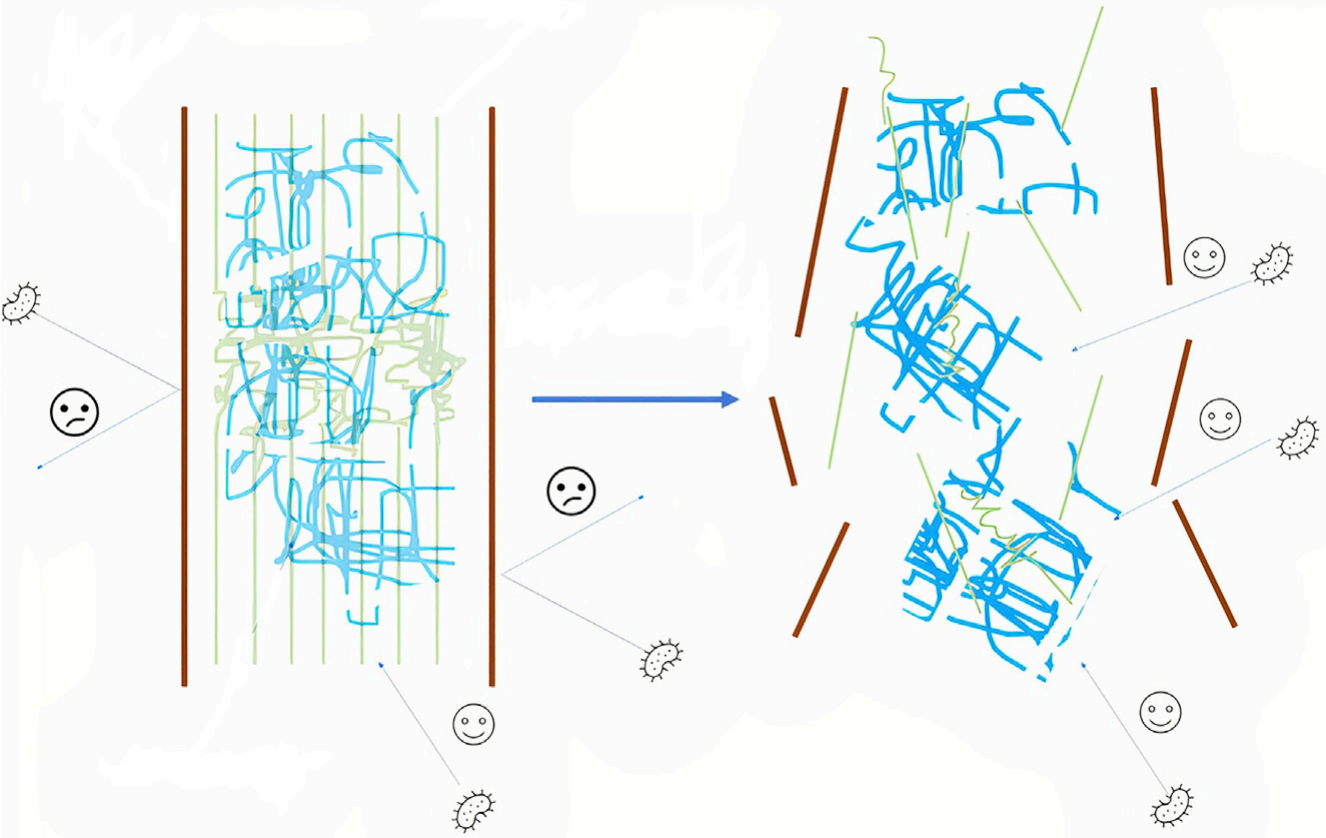
Effect of mechanical treatments on LCB biomass, from left (raw material) to right (mechanically treated material) Brown lines represent lignin, green lines represent cellulose, and blue lines represent hemicellulose.

As shown in Figure 13, enzymatic and fermentative processes accounted for 47% and 15% of the revised articles, respectively. However, there are also other options so that biomass could be valorized. For example, the chemical path (23% of the articles consulted chose this path) aims to obtain fine chemicals from the hemicellulose and cellulose sugars or aromatic compounds from lignin. Shen et al. (2020) used mechanochemical treatments to increase the production of 5-Hydroxymethylfurfural from cellulose (Shen et al., 2020). Other authors implemented mechanical treatments to increase the amount of silica recovered from LCB ashes (Park et al., 2021). Thermal treatments represented 9% of the references consulted, including technologies such as pyrolysis or microwave (Mayer-Laigle et al., 2020; Yang et al., 2022). Finally, other treatments represented 6% of the references consulted and focused on the obtention of micro and nano cellulose (Ämmälä et al., 2019; Ferreira et al., 2020). However, enzymatic and fermentative are the most used among the references studied. This review focuses on these valorization paths. Figure 14 shows how mechanical treatments improve the effectiveness of enzymes.

As can be seen from the previous illustration, mechanical treatments break down the cell wall to expose fibers from the lignin-cellulose complex making it more accessible for microorganisms or enzymes.

### Enzymatic treatment

Enzymes act as catalyzers, increasing the rate of biological reactions by decreasing the activation energy under mild conditions. Depending on the reaction they catalyze, there are several enzymes: Oxidoreductase, transferases, hydrolases, lyases, ligases, and isomerases (Blanco and Blanco, 2017).

When used for the treatment of LCB, enzymes usually focus on the cleavage links of cellulose/hemicellulose, resulting in simpler molecules, so the efficiency of the following process is increased. However, using enzymes on raw LCB usually gives poor performance (Maitra and Singh, 2021) because of the recalcitrant characteristics of this material. Therefore, LCB needs treatment to increase the performance of enzymes. Mechanical treatments do not inhibit the activity of enzymes as they disrupt the molecules mechanically and do not change the molecules chemically. Furthermore, mechanical treatments have the highest mass yield of all the pretreatments.

Sitotaw et al. (2022) studied the possibility of using mechanochemical treatments to improve the performance of enzymatic treatments. It was discovered that mechanical treatments improve cellulose release compared with untreated material. Furthermore, the mechanochemical approach improved sugar yield by up to 86%, which was quicker (Sitotaw et al., 2022). Other researchers used mechanical treatments to increase corn stover saccharification by thread rolling. It was found that this process can increase enzymatic activity. Bigger particles had lower efficiency regarding saccharification (17.5% sugar release for 20-40 mesh) than smaller particles (50.69% sugar release for 60-80 mesh) (Deng and Li, 2021). However, enzymatic hydrolysis is not only used for saccharification or glucose release. This method can also be applied to dissolving pulp to increase the accessibility of cellulose. As dissolving pulp is high-purity cellulose, it also has high crystallinity, which hinders the action of chemicals from modifying this structure. According to Wang et al.(2020), mechanical treatments coupled with enzymes can reduce crystallinity from 68.8 to 47.1% leading to an increase in Fock’s reactivity (which is related to the consumption of CS_2_ during viscose production) from 54.8 to 78% (Wang et al., 2020). However, performing an enzymatic treatment on LCB is not enough for the residues to be valorized. Therefore, there must be a definitive treatment for the LCB to be valorized, and this treatment is usually the fermentation of the released sugars.

### Fermentative treatments

Fermentative treatments use microorganisms (bacteria or fungi) to valorize LCB through anaerobic digestion (A.D.). These microorganisms use carbohydrates from LCB as substrate, and, as a result, products are obtained. The main advantages of this process are the low energy consumption and the low waste generation (Llano et al., 2021). However, it needs large equipment and high residence times to complete the reaction (Amin et al., 2017). Again, owing to the recalcitrant nature of LCB, A.D. is usually performed with previous treatments, so reducing sugars can become more accessible (Luo et al., 2021). Authors have used mechanical treatments before A.D., i.e., Garuti et al. (2022) analyzed several size reductions equipment to calculate the efficiency of the process regarding energy. After mechanical treatments, it was found that methane yield increased from 1% to 13%, and the maximum methane production increased from 4% to 48%. Additionally, every equipment used for size reduction led to a positive energy balance (Garuti et al., 2022).

Generally, A.D. aims to obtain methane or biogas. However, other authors used A.D. to produce hydrogen by photofermentative bacteria. Corn stover was used as the substrate, and it was pretreated with ultrafine grinding. It was found that increasing grinding time improved H_2_ yield and reduced residence time from 36 to 24 h (Tahir et al., 2021). Navarro-Mtz et al., 2019 used soybean meal, a co-product after extracting oil, after mechanical treatment as culture media for microorganism culture (*Bacillus thuringiensis*). Mechanical treatment led to an increase of released sugar by 34.1 and 2.5 times more fermentable sugars when compared to untreated texture soybean and commercial soybean meal, respectively. Cell growth also was higher than standard culture media without the generation of inhibitors (Navarro-Mtz et al., 2019).

## Conclusions

This review showed recent research on mechanical treatments, focusing on posterior enzymatic/fermentative treatments. Mechanical treatments effectively release cellulose chains within the LCB structure by physically exposing the fibers without altering their chemical properties. In the literature, enzymatic and fermentative treatments have been extensively used as a final treatment after mechanical treatments. However, it must be noted that these treatments need to be controlled in terms of energy to be economically and environmentally feasible. To overcome this significant drawback, optimizing the energetic requirements to a specific particle size should be performed. This optimization should be performed for each feedstock and mill as biomass feedstock is very heterogeneous in chemical and mechanical properties.

Additionally, selecting the proper size reduction mechanism depending on the feedstock is critical as it can lead to excessive energy use. Ball mill is, by far, the most used size reduction operation when working with LCB because it can lead to smaller sizes and is easy to operate. However, as it is a batch operation, scaling it up to an industrial scale needs extra work than continuous comminution processes. In this sense, knife or hammer mills are more suitable for scaling up to an industrial scale. Target particle size is 1 mm, and energy requirements are generally lower when compared to ball milling. Fermentative and enzymatic treatments are also eco-friendly as no waste is generated. Furthermore, if used to produce biogas or hydrogen, it would help to ameliorate greenhouse gas emissions.
